# Short-term and long-term comparisons of laparoscopy-assisted proximal gastrectomy with esophagogastrostomy by the double-flap technique and laparoscopy-assisted total gastrectomy for proximal gastric cancer

**DOI:** 10.1371/journal.pone.0242223

**Published:** 2020-11-12

**Authors:** Tomoko Tsumura, Shinji Kuroda, Masahiko Nishizaki, Satoru Kikuchi, Yoshihiko Kakiuchi, Nobuo Takata, Atene Ito, Megumi Watanabe, Kazuya Kuwada, Shunsuke Kagawa, Toshiyoshi Fujiwara

**Affiliations:** 1 Department of Gastroenterological Surgery, Okayama University Graduate School of Medicine, Dentistry and Pharmaceutical Sciences, Okayama, Japan; 2 Center for Innovative Clinical Medicine, Okayama University Hospital, Okayama, Japan; 3 Minimally Invasive Therapy Center, Okayama University Hospital, Okayama, Japan; 4 Department of Surgery, Tsuyama Chuo Hospital, Tsuyama, Japan; 5 Department of Surgery, Iwakuni Clinical Center, Iwakuni, Japan; 6 Department of Gastroenterological Surgery, Japanese Red Cross Okayama Hospital, Okayama, Japan; Clinic for Infectious and tropical diseases, Clinical centre of Serbia, SERBIA

## Abstract

**Background:**

Although proximal gastrectomy (PG) is a recognized surgical procedure for early proximal gastric cancer, total gastrectomy (TG) is sometimes selected due to concern about severe gastroesophageal reflux. Esophagogastrostomy by the double-flap technique (DFT) is an anti-reflux reconstruction after PG, and its short-term effectiveness has been reported. However, little is known about the long-term effects on nutritional status and quality of life (QOL).

**Methods:**

Gastric cancer patients who underwent laparoscopy-assisted PG (LAPG) with DFT or laparoscopy-assisted TG (LATG) between April 2011 and March 2014 were retrospectively analyzed. Body weight (BW), body mass index (BMI), and prognostic nutritional index (PNI) were reviewed to assess nutritional status, and the Postgastrectomy Syndrome Assessment Scale (PGSAS)-45 was used to assess QOL.

**Results:**

A total of 36 patients (LATG: 17, LAPG: 19) were enrolled. Four of 17 LATG patients (24%) were diagnosed with Stage ≥II after surgery, and half received S-1 adjuvant chemotherapy. BW and PNI were better maintained in LAPG than in LATG patients until 1-year follow-up. Seven of 16 LATG patients (44%) were categorized as “underweight (BMI<18.5 kg/m^2^)” at 1-year follow-up, compared to three of 18 LAPG patients (17%; *p* = 0.0836). The PGSAS-45 showed no significant difference in all QOL categories except for decreased BW (*p* = 0.0132). Multivariate analysis showed that LATG was the only potential risk factor for severe BW loss (odds ratio: 3.03, *p* = 0.0722).

**Conclusions:**

LAPG with DFT was superior to LATG in postoperative nutritional maintenance, and can be the first option for early proximal gastric cancer.

## Introduction

Proximal gastrectomy (PG) is described in the Japanese gastric cancer treatment guideline as a surgical procedure that can be considered for proximal tumors where more than half of the distal stomach can be preserved [1]. Though PG is preferable in terms of organ preservation and function preservation, no standard reconstruction method after PG has yet been established, mainly due to the problem of gastroesophageal reflux after surgery [2]. Esophagogastrostomy (EG) with no additional anti-reflux procedure often causes severe reflux esophagitis, and even alternative procedures, such as jejunal interposition (JI), jejunal pouch interposition (JPI), and a double-tract (DT), do not resolve this problem satisfactorily. Unsuccessful reconstruction after PG causes substantial decline in quality of life (QOL) after surgery [3], and for this reason, total gastrectomy (TG) is sometimes selected as the first option at the expense of stomach preservation, although the prognosis after PG is reportedly comparable to that after TG [4, 5].

Our first-choice reconstruction method for proximal gastric cancer is PG with the double-flap technique (DFT) reconstruction. DFT reconstruction, also known as the Kamikawa procedure, is an anti-reflux EG after PG, first reported by Kamikawa et al in 1998, in which the distal esophagus and the anastomosis are embedded into the submucosal space of the gastric remnant and covered by the seromuscular double-flap [6, 7]. While DFT reconstruction has been reported to show acceptable short-term outcomes in preventing reflux esophagitis after surgery in a multicenter, retrospective study [8], there is little evidence of the long-term outcomes, including patients’ QOL.

In the present study, the feasibility of laparoscopy-assisted PG (LAPG) with DFT reconstruction was compared with that of laparoscopy-assisted TG (LATG) in terms of short-term and long-term effects on body weight (BW) and QOL after surgery. This study will help establish a standard surgical procedure for early proximal gastric cancer.

## Methods

### Patients

The medical records of patients with gastric cancer who underwent LAPG with D1+ lymph node dissection followed by DFT reconstruction or who underwent LATG with D1+ lymph node dissection followed by Roux-en-Y reconstruction between April 2011 and March 2014 at Okayama University Hospital were reviewed. This study was reviewed and approved by the institutional review board (IRB) of Okayama University Hospital (#1505–022). The IRB waived the requirement to obtain informed consent for this study.

### Surgical procedures of LATG and LAPG

In both LATG and LAPG, gastrectomy and lymph node dissection were performed laparoscopically in a conventional 5-port setting, and reconstruction was performed under direct vision through a small incision placed in the epigastrium. Esophago-jejunal anastomosis and jejuno-jejunal anastomosis in LATG were conducted using a circular stapler and a linear stapler, respectively. DFT reconstruction was conducted by a complete hand-sewn suturing process, as previously reported [6].

### Clinical data

Patients’ characteristics included age, sex, height, BW, body mass index (BMI), preoperative co-morbidities, American Society of Anesthesiologists physical status (ASA-PS) classification, vital capacity (VC), forced expiratory volume 1.0 (FEV1.0), prognostic nutritional index (PNI) and presence or absence of sarcopenia. Histological findings such as histological type, pathological depth of tumor (pT), pathological lymph node metastasis (pN), and pathological stage (pStage) were described according to the 3rd English edition of the Japanese Classification of Gastric Carcinoma [9]. Surgical outcomes included operation time, blood loss, length of skin incision, presence or absence of concurrent cholecystectomy, postoperative complications classified according to the Clavien-Dindo classification, highest body temperature (BT) after surgery, duration of BT ≥37.5°C, the number of days until the first flatus after surgery, and the length of hospital stay after surgery. BW was recorded at 1-month, 6-month, and 1-year follow-ups, and PNI was recorded at 6-month and 1-year follow-ups as well. At 1-year follow-up, reflux esophagitis was evaluated by endoscopic examination and classified according to the Los Angeles (LA) classification [10].

### QOL survey

At approximately 3 years after surgery, a QOL survey was carried out by mail using the questionnaire of the Postgastrectomy Syndrome Assessment Scale (PGSAS)-45, which consists of a total of 45 questions [11, 12]. Based on this questionnaire, the total symptom score and the 7 symptom subscales (SS) for esophageal reflux, abdominal pain, meal-related distress, indigestion, diarrhea, constipation, and dumping were calculated. In addition, decreased BW, ingested amount of food per meal, need for additional meals, quality of ingestion, ability to work, dissatisfaction with symptoms, dissatisfaction with meals, dissatisfaction with working, dissatisfaction with daily life, physical component summary, and mental component summary were also assessed. PGSAS-45 was proved to be a valid and reliable questionnaire for assessment of living status and QOL in postgastrectomy patients in a previous study [11].

### Statistical analysis

Statistical analysis was conducted using JMP software (SAS Institute, Cary, NC, USA). Student’s *t-*test was used to assess the continuous variables of age, BW, BMI, VC, FEV1.0, and PNI. The Wilcoxon signed-rank test was used for the other continuous variables of operation time, blood loss, length of skin incision, highest BT, duration of BT ≥37.5°C, the number of days until the first flatus after surgery, and the length of hospital stay after surgery. Pearson’s χ^2^ test was used for the categorical variables of sex, presence of preoperative co-morbidities, ASA-PS, histological type, pT, pN, pStage, concurrent cholecystectomy, postoperative complications, and BMI <18.5 kg/m^2^. QOL was also compared by the Wilcoxon signed-rank test. Overall survival and recurrence-free survival curves were evaluated using the Kaplan-Meier method. A *p* value <0.05 was considered significant.

## Results

### Patients’ characteristics

**Fig 1:**
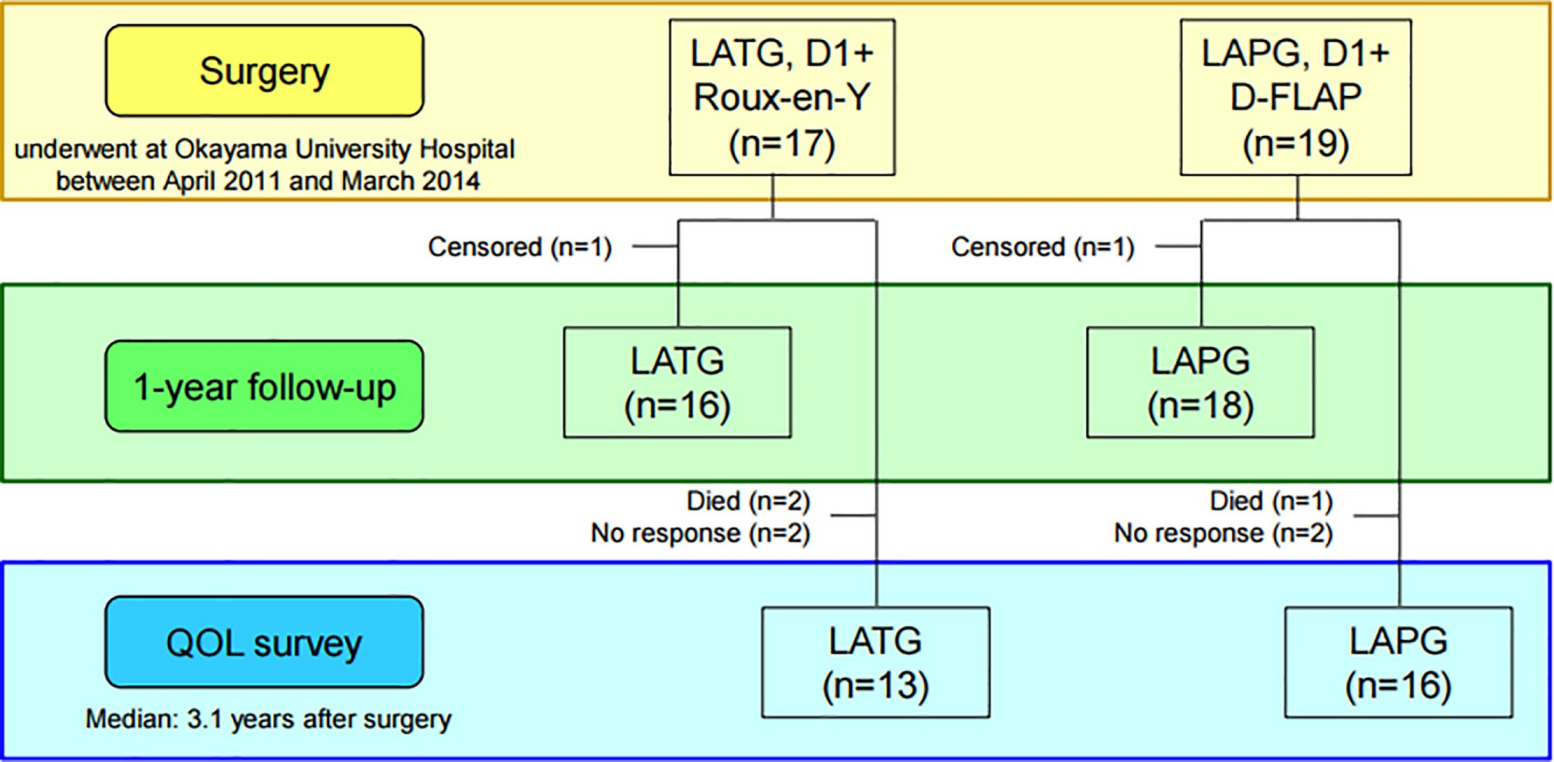
CONSORT diagram.

**Fig 2:**
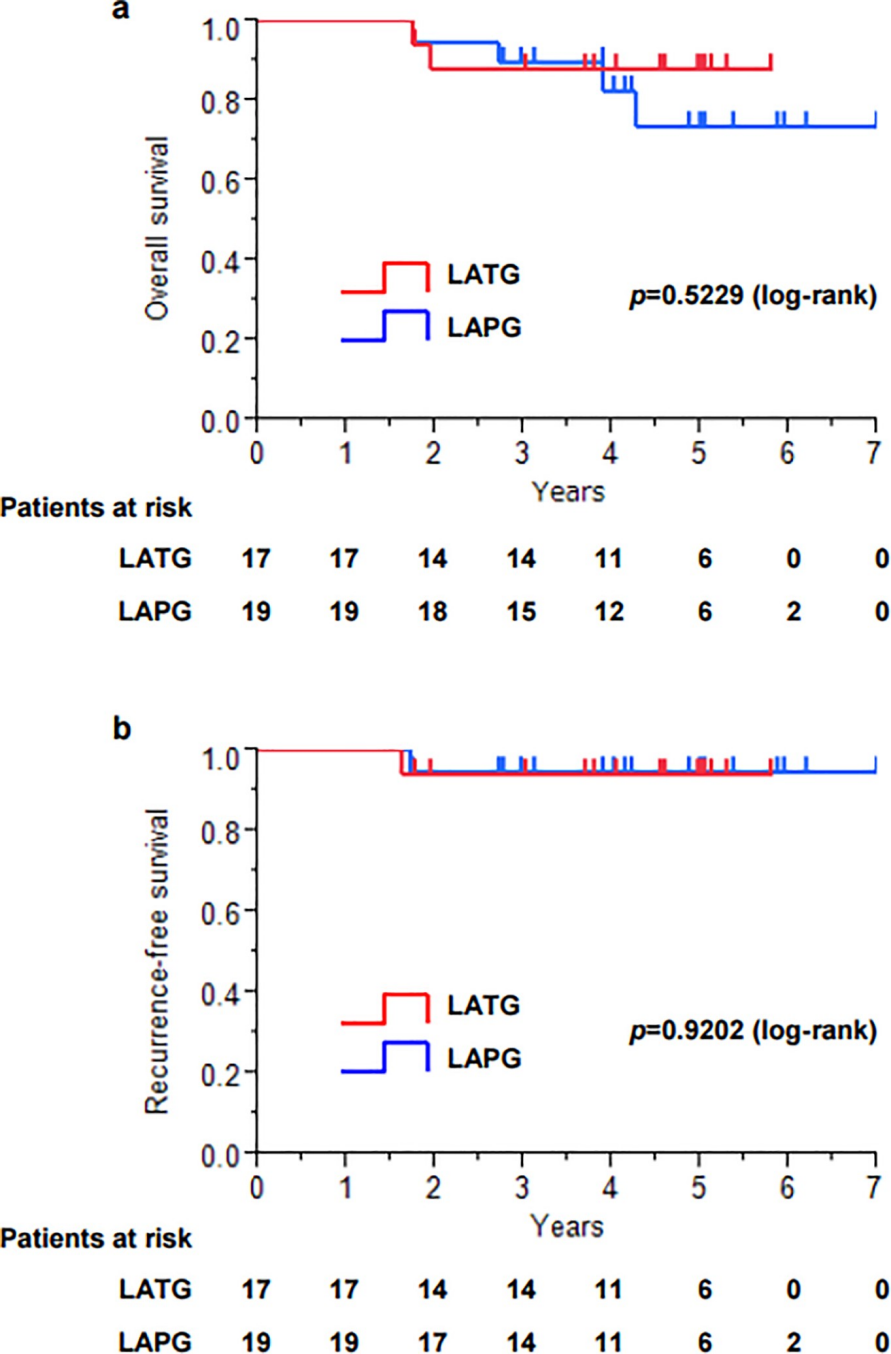
Kaplan-Meier survival analysis. a. Overall survival. b. Recurrence-free survival.

A total of 36 patients were enrolled, 17 patients who underwent LATG and 19 patients who underwent LAPG (Fig 1). In the patients’ characteristics and histological findings (Table 1), there were no significant differences between LATG and LAPG except for histological type and pStage. The proportion of undifferentiated type was significantly higher in LATG (44%) than in LAPG (5%) (*p* = 0.0069). All 19 patients who underwent LAPG were diagnosed with pStage I after surgery, whereas four of 17 patients (24%) who underwent LATG were diagnosed with pStage II or higher after surgery, although all 17 patients were diagnosed with clinical T1 and N0 before surgery, and 2 of the 4 patients received adjuvant chemotherapy with S-1 after surgery according to the Japanese gastric cancer treatment guidelines 2010 (ver.3) [1]. The Kaplan-Meier survival analysis (median follow-up period, 4.2 years) showed that there were no significant differences between LATG and LAPG in overall survival (*p* = 0.5229) and recurrence-free survival (*p* = 0.9202) (Fig 2).

**Table 1:** Patients’ characteristics and histological findings.

	LATG (n = 17)	LAPG (n = 19)	p value
Age, y			
Mean ± SD	68.9 ± 12.1	71.2 ± 8.0	0.5063
Sex			
Male	11 (65%)	16 (84%)	0.1773
Female	6 (35%)	3 (16%)	
BMI, kg/m^2^			
Mean ± SD	23.6 ± 3.3	23.5 ± 3.5	0.9363
Preoperative co-morbidities			
Yes	11 (65%)	15 (79%)	0.3409
ASA-PS			
1	3 (18%)	2 (11%)	0.8266
2	9 (53%)	11 (58%)	
≥3	5 (29%)	6 (32%)	
VC, %			
Mean ± SD	105.4 ± 18.2	99.5 ± 18.2	0.3390
FEV1.0, %			
Mean ± SD	73.1 ± 9.3	73.1 ± 10.9	0.9925
PNI			
Mean ± SD	51.3 ± 6.0	51.1 ± 5.6	0.9049
Sarcopenia			
Yes	3 (20%)	4 (24%)	0.8096
Histological type			
Differentiated	9 (56%)	18 (95%)	0.0069
Undifferentiated	7 (44%)	1 (5%)	
Pathological T status (pT)			
1	14 (82%)	17 (89%)	0.5374
≥2	3 (18%)	2 (11%)	
Pathological N status (pN)			
0	14 (82%)	18 (95%)	0.2379
≥1	3 (18%)	1 (5%)	
Pathological Stage (pStage)			
IA	13 (76%)	16 (84%)	0.0270
IB	0 (0%)	3 (16%)	
≥II	4 (24%)	0 (0%)	

BMI, body mass index; VC, vital capacity; FEV1.0, forced expiratory volume 1.0; PNI, prognostic nutritional index; SD, standard deviation.

### Surgical outcomes

In surgical outcomes (Table 2), operation time was not significantly different between LATG (306 min) and LAPG (280 min) (*p* = 0.6804), whereas blood loss was significantly higher in LAPG (210 mL) than in LATG (70 mL) (*p* = 0.0106). The skin incision placed in the epigastrium for reconstruction was significantly longer in LAPG (8 cm) than in LATG (7 cm) (*p* = 0.0349), which may have been influenced by complexity of reconstruction procedure. There was no significant difference in the incidence of postoperative complications (*p* = 0.9060). In detail, the incidence of postoperative complications (CD any grade) in LATG was 12% (two of 17 patients) including an anastomotic leakage (5%), while that in LAPG was 11% (two of 19 patients), including an anastomotic stricture (5%). In the postoperative courses, there were no significant differences except for the number of days until the first flatus after surgery, which was significantly higher in LATG (2 days) than in LAPG (1 day) (*p* = 0.0168). This meant earlier recovery of bowel function in LAPG and may have led to shorter hospital stay in LAPG (12 days) than in LATG (13 days) although difference was not statistically significant (p = 0.1241).

**Table 2:** Surgical outcomes.

	LATG (n = 17)	LAPG (n = 19)	p value
Operation time, min			
Median (IQR)	306 (256.5–371.5)	280 (264–345)	0.6804
Blood loss, mL			
Median (IQR)	70 (40–117.5)	210 (90–285)	0.0106
Skin incision, cm			
Median (IQR)	7 (6–8)	8 (7–10)	0.0349
Concurrent cholecystectomy			
Yes	2 (12%)	2 (11%)	0.9060
Postoperative complications			
Any Grade^a^	2 (12%)	2 (11%)	0.9060
Anastomotic leakage	1 (5%)	0 (0%)	
Anastomotic stricture	0 (0%)	1 (5%)	
Pneumonia	0 (0%)	1 (5%)	
Urinary tract infection	1 (5%)	0 (0%)	
Highest BT, ^o^C			
Median (IQR)	38.0 (37.5–38.65)	37.9 (37.5–38.2)	0.6913
Duration of BT ≥37.5 ^o^C			
Median (IQR)	1 (0.5–3)	1 (0–2)	0.3554
First flatus, POD			
Median (IQR)	2 (2–3)	1 (1–2)	0.0168
Hospital stay, days			
Median (IQR)	13 (11.5–15)	12 (11–14)	0.1241

BT, body temperature; POD, postoperative day; IQR, interquartile range.

^a^ according to the Clavien-Dindo classification.

### One-year follow-up

**Fig 3:**
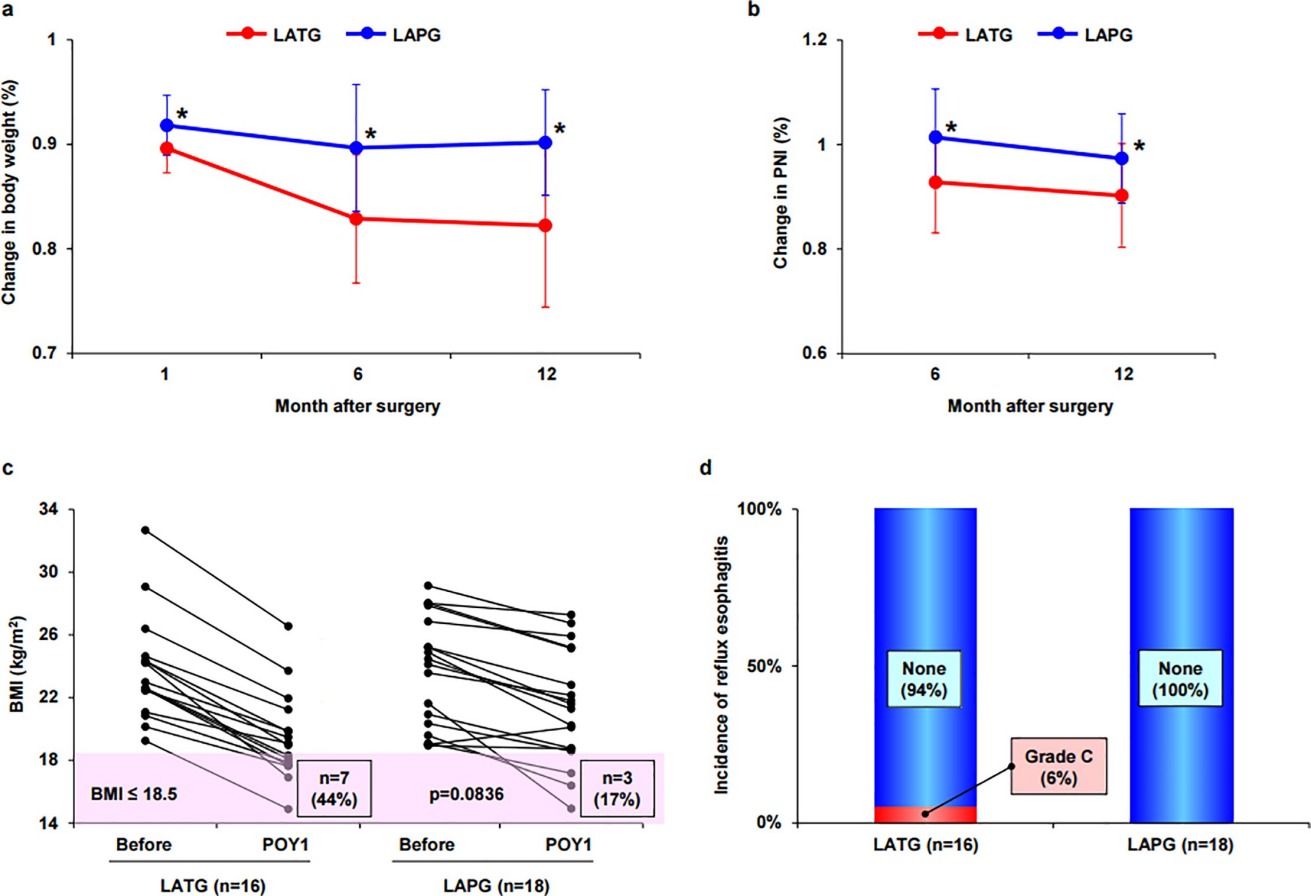
Follow-up to one year after surgery. a. Change in body weight. *, *p*<0.05. b. Change in PNI. *, *p*<0.05. c. Change in BMI. BMI ≤ 18.5 kg/m^2^ is defined as “underweight”. POY, postoperative year. d. Incidence of reflux esophagitis.

Overall, 16 patients who underwent LATG and 18 patients who underwent LAPG were monitored for at least until one year after surgery (Fig 1). BW change at 1, 6, and 12 months after surgery (LATG vs LAPG) was -10.4% vs -8.2%, -17.1% vs -10.4%, and -17.8% vs -9.8%, respectively; BW loss was significantly smaller in LAPG at every time point (Fig 3A). The same applied to the change in PNI. PNI change at 6 and 12 months after surgery (LATG vs LAPG) was -7.3% vs +1.4% and -9.8% vs -3.7%, respectively; PNI was significantly higher in LAPG at every time point (Fig 3B). When BMI<18.5 kg/m^2^ was defined as “underweight”, seven of 16 LATG patients (44%) and three of 18 LAPG patients (17%) were categorized as “underweight” at 1-year follow-up (*p* = 0.0836), though no patient in both procedures was categorized as “underweight” before surgery (Fig 3C). Endoscopic examination at 1-year follow-up showed that no LAPG patient had reflux esophagitis, whereas one LATG patient had reflux esophagitis (LA grade C) (Fig 3D).

### Postoperative QOL assessment with the PGSAS-45

Twenty-nine of 36 patients responded to the QOL survey by mail (collection rate, 81%), including 13 of 17 LATG patients and 16 of 19 LAPG patients in, and the median time after surgery of these patients at the time of this QOL survey was 3.1 years (Fig 1). There were no significant differences in all categories between LATG and LAPG patients except for decreased BW (*p* = 0.0132), which was 15.1% in LATG patients and 9.6% in LAPG patients (Table 3). The esophageal reflux SS of LAPG was 1.3, which was as good as the 1.5 of LATG. When BW loss ≥15% was defined as “severe BW loss”, univariate and multivariate analyses showed that LATG was a potential risk factor for “severe BW loss” (odds ratio: 3.03, *p* = 0.0722) (Table 4).

**Table 3:** Postoperative QOL assessment with PGSAS-45.

		LATG (n = 13)		LAPG (n = 16)		p value
		Median	IQR	Median	IQR	
Symptoms						
	Esophageal reflux	1.5	(1.1–2.5)	1.3	(1.0–2.4)	0.3531
	Abdominal pain	1.3	(1.0–1.7)	1.7	(1.0–2.2)	0.5112
	Meal-related distress	2.7	(2.0–4.0)	2.3	(1.7–2.7)	0.2509
	Indigestion	2.0	(1.8–2.6)	1.9	(1.0–2.7)	0.6419
	Diarrhea	2.7	(1.5–3.7)	2.0	(1.2–3.3)	0.5511
	Constipation	2.0	(1.3–2.8)	2.5	(1.8–3.3)	0.2607
	Dumping	2.0	(1.0–2.7)	1.0	(1.0–2.1)	0.1999
	Total symptom	1.9	(1.7–2.8)	1.9	(1.5–2.4)	0.6930
Living status						
	Decrease in body weight (%)	15.1	(14.5–24.6)	9.6	(5.0–14.4)	0.0132
	Ingested amount of food per meal	6.0	(5.0–8.0)	6.5	(5.0–8.0)	0.5314
	Necessity for additional meals	2.0	(1.5–2.5)	2.0	(1.0–2.0)	0.3275
	Quality of ingestion	4.0	(3.3–4.7)	3.8	(3.0–4.9)	0.7557
	Ability for work	2.0	(1.5–2.5)	2.0	(1.0–3.8)	0.6313
QOL						
	Dissatisfaction with symptoms	1.0	(1.0–2.0)	1.0	(1.0–1.8)	0.5368
	Dissatisfaction at the meals	3.0	(1.0–3.0)	3.0	(1.0–3.0)	0.6005
	Dissatisfaction at working	1.0	(1.0–2.0)	1.5	(1.0–3.0)	0.4388
	Dissatisfaction for daily life	2.0	(1.0–2.5)	1.5	(1.0–2.8)	0.9283
	Physical component summary	52.2	(49.5–52.9)	47.9	(41.4–54.3)	0.1604
	Mental component summary	51.6	(44.5–55.2)	53.2	(45.6–54.9)	0.9650

**Table 4:** Univariate and multivariate analyses of risk factors for severe body weight loss.

		Univariate	Multivariate	
		*p* value	OR	*p* value
**Background**	Age (≥80 y)	0.3414		
	Sex (Male)	0.1552		
	BMI (≥25 kg/m^2^)	0.0795	0.71	0.3872
	Comorbidity (+)	0.1343		
	ASA-PS (≥3)	0.7394		
	PNI (<50)	0.2920		
	Sarcopenia (+)	0.9399		
	Pathological Stage (pStage ≥II)	0.5714		
**Operation**	Operation procedure (LATG)	0.0462	3.03	0.0722
	Operation time (≥360 min)	0.7064		
	Blood loss (≥300 mL)	0.6569		
	Postoperative complications	0.4911		
	Clavien-Dindo (≥II)	1.0000		
Postoperative chemotherapy (+)		0.6402		

BMI, body mass index; PNI, prognostic nutritional index; CI, confidence interval; OR, odds ratio.

## Discussion

In the present study, the advantages of LAPG with DFT over LATG were evaluated in terms of short-term and long-term nutritional maintenance. BW and PNI were actually better maintained after LAPG with DFT than after LATG until 1-year follow up, which was consistent with many previous reports. Sugiyama et al reported that laparoscopic PG (LPG) with DT reconstruction maintained BW and skeletal muscle better than laparoscopic TG (LTG) at 1 year after surgery [13], and Kosuga et al reported that nutritional status, such as changes in BW and blood chemistries including hemoglobin, serum albumin, and total lymphocyte count, was consistently better in LPG with EG than LTG at 6 months and 1 and 2 years after surgery [14]. BMI is a simple value calculated from weight and height, and it is a convenient measure representing nutritional status. BMI <18.5 kg/m^2^ is defined as “underweight” in the World Health Organization (WHO) criteria, and being “underweight” is in general associated with a variety of health risks. In gastric cancer as well, “underweight” patients were reported to have the worst overall survival and disease-specific survival among six categories (“underweight”, “normal-weight”, “overweight”, “mildly obese”, “moderately obese”, and “severely obese”) divided according to BMI [15]. In the present study, seven of 16 patients (44%) were “underweight” 1 year after LATG, though no patient was “underweight” before surgery. Two patients died after LATG in the present study, both of whom were “underweight” 1 year after surgery, and the causes of death were gastric cancer death and multiple organ failure. In contrast, only three of 18 patients (17%) were “underweight” 1 year after LAPG, but one of 4 patients who died after LAPG was “underweight” 1 year after surgery, and the cause of death was pneumonitis. Based on these results, severe BW loss, especially to BMI <18.5 kg/m^2^, is critical, and, therefore, PG, which can maintain BW better than TG, will be recommended for early proximal gastric cancer from the standpoint of nutritional maintenance and health risks.

However, to select PG as a standard procedure for early proximal gastric cancer, a reconstruction method that can effectively maintain the patients’ postoperative QOL is definitely necessary. DFT, one of the EGs with an anti-reflux procedure, has recently been becoming increasingly commonly performed in Japan based on its capability of effectively preventing gastroesophageal reflux after surgery, leading to a large decline in postoperative QOL [16]. In the present study, reflux esophagitis was not observed in any patients on endoscopic examination at 1-year follow-up after LAPG with DFT, whereas the incidence of reflux esophagitis (LA grade B or higher) after DFT investigated in a previous multicenter retrospective study was 6.0%, which was considered acceptable [8]. The PGSAS-45 questionnaire, in which the severity of gastroesophageal reflux is assessed by the esophageal reflux SS, was used for the QOL survey in the present study. The range of this scale is 1.0 to 5.0, and a smaller value means less symptoms. In the present study, the esophageal reflux SS of LAPG was 1.3, which was considered relatively good and comparable to that of LATG, in which esophageal reflux is fundamentally unlikely to happen. When the present data of the esophageal reflux SS of LAPG were compared with the previous national multi-institutional data of this scale of the PGSAS-45 after PG [17], in which 115 cases of EG, 34 cases of JI, and 44 cases of JPI were included, by using the PGSAS Statistics Kit available on the web, the esophageal reflux SS of the present study (mean: 1.6, standard deviation [SD]: 0.8) was better than that of the national data (mean: 2.0, SD: 1.0) (*p* = 0.063, Cohen’s *d* = 0.41) [18], which may show that DFT has some advantage over a variety of reconstruction methods after PG.

While the present study produced some interesting outcomes, it does have several limitations. First, this was a retrospective, single-center study with a limited number of cases. Second, four LATG patients were diagnosed with advanced stage gastric cancer, and two of them received adjuvant chemotherapy with S-1 for a year after surgery, which may have affected postoperative nutritional status. Third, nutritional status was evaluated only by BW and PNI, and other major nutritional indices, such as the Glasgow Prognostic Score (GPS), the neutrophil/lymphocyte ratio (NLR), and Controlling Nutritional Status (CONUT score), were not assessed in this study. Then, considering the surgical outcomes, the reason why blood loss was significantly higher in LAPG than in LATG was actually hard to explain, but one possibility might be that gastric fluid overflowing during the DFT procedure was counted as blood loss.

Although a comparison of DFT with other EGs or other types of reconstruction methods such as DT was the most intriguing topic in the evaluation of appropriate reconstruction methods after PG, the present study demonstrated that LAPG with DFT was superior at least to LATG in terms of short-term and long-term nutritional maintenance after surgery, without decreasing QOL. Focusing on BW loss after surgery, this study showed that TG was associated with a higher risk of “underweight” (BMI <18.5 kg/m^2^) after surgery, which leads to overall health risks irrespective of cancer, and was the only potential risk factor for severe BW loss (>15%) on univariate and multivariate analyses. In conclusion, LAPG with DFT reconstruction can be the first option for early gastric cancer located in the proximal stomach from a standpoint of postoperative nutritional maintenance although QOL may not be much different from LATG.
